# Toward integrative cancer immunotherapy: targeting the tumor microenvironment

**DOI:** 10.1186/1479-5876-10-70

**Published:** 2012-04-10

**Authors:** Leisha A Emens, Samuel C Silverstein, Samir Khleif, Francesco M Marincola, Jérôme Galon

**Affiliations:** 1 Tumor Immunology and Breast Cancer Research Programs, Johns Hopkins University School of Medicine, Baltimore, MD 21231, USA; 2 Department of Oncology, Johns Hopkins University School of Medicine, Baltimore, MD 21231, USA; 3 Department of Physiology and Cellular Biophysics, Columbia University College of Physicians and Surgeons, New York, NY 10032, USA; 4 Department of Medicine, Columbia University College of Physicians and Surgeons, New York, NY 10032, USA; 5 Georgia Health Sciences University (GHSU) Cancer Center, Georgia, USA; 6 Infectious Diseases and Immunogenetics Section (IDIS), National Institutes of Health, Bethesda, MD 20891, USA; 7 Department of Transfusion Medicine, Clinical Center and trans-NIH Center for Human Immunology (CHI), National Institutes of Health, Bethesda, MD 20891, USA; 8 INSERM, UMRS872, Cordeliers Research Centre, Laboratory of Integrative Cancer Immunology, Paris F-75006, France; 9 Assistance Publique-Hopitaux de Paris, AP-HP, Georges Pompidou European Hospital, Paris, France; 10 Université Paris Descartes, Paris, France; 11 Université Pierre et Marie Curie Paris 6, Paris, France; 12 The Sidney Kimmel Comprehensive Cancer Center at Johns Hopkins, Johns Hopkins University, 1650 Orleans Street, Room 409, Bunting Blaustein Cancer Research Building, Baltimore, MD 21231-1000, USA

## Abstract

The development of cancer has historically been attributed to genomic alterations of normal host cells. Accordingly, the aim of most traditional cancer therapies has been to destroy the transformed cells themselves. There is now widespread appreciation that the progressive growth and metastatic spread of cancer cells requires the cooperation of normal host cells (endothelial cells, fibroblasts, other mesenchymal cells, and immune cells), both local to, and at sites distant from, the site at which malignant transformation occurs. It is the balance of these cellular interactions that both determines the natural history of the cancer, and influences its response to therapy. This active tumor-host dynamic has stimulated interest in the tumor microenvironment as a key target for both cancer diagnosis and therapy. Recent data has demonstrated both that the presence of CD8^+ ^T cells within a tumor is associated with a good prognosis, and that the eradication of all malignantly transformed cells within a tumor requires that the intra-tumoral concentration of cytolytically active CD8^+ ^effector T cells remain above a critical concentration until every tumor cell has been killed. These findings have stimulated two initiatives in the field of cancer immunotherapy that focus on the tumor microenvironment. The first is the development of the immune score as part of the routine diagnostic and prognostic evaluation of human cancers, and the second is the development of combinatorial immune-based therapies that reduce tumor-associated immune suppression to unleash pre-existing or therapeutically-induced tumor immunity. In support of these efforts, the Society for the Immunotherapy of Cancer (SITC) is sponsoring a workshop entitled "Focus on the Target: The Tumor Microenvironment" to be held October 24-25, 2012 in Bethesda, Maryland. This meeting should support development of the immune score, and result in a position paper highlighting opportunities for the development of integrative cancer immunotherapies that sculpt the tumor microenvironment to promote definitive tumor rejection.

## Introduction

Historically, cancer therapies have largely focused on destroying the transformed cancer cell itself. Local therapies, including surgery and radiotherapy, aim to grossly neutralize malignancy, either by removing the tumor, or by destroying the replicative capacity of the cancer cells within it. Chemotherapy (and for some cancers endocrine therapy) classically exerts an anti-tumor effect by selectively disrupting an aspect of tumor cell biology that gives malignant cells a relative growth advantage compared to normal cells. More recently, innovative targeted therapies have been developed that selectively target and disrupt signaling pathways essential for tumor cell growth; examples include the HER-2-specific monoclonal antibody Trastuzumab for breast cancer, and the BRAF inhibitor vemurafenib for melanoma. Their use results in higher cure rates and less collateral damage to normal tissues than conventional chemotherapy or radiotherapy. Effectively sequencing these different treatment modalities and using combinations of drugs with complementary mechanisms of action and non-overlapping toxicities has reduced the mortality rates for many cancers [1].

There is now widespread appreciation that non-transformed host cells (endothelial cells, fibroblasts, other mesenchymal cells, and cells of the innate and adaptive immune systems) interact with malignant tumor cells to form a dynamic tumor microenvironment in which the non-transformed cells exert both positive and negative effects on the growth and spread of the cancer cells, and that these in turn affect the phenotype of the non-transformed host cells [2]. The balance of cellular and secretory-product interactions within this microenvironment determines whether the tumor mass regresses or grows, and whether the malignant cells remain in place, or metastasize to distant sites. In addition, these interactions can determine whether tumors respond well to radiation or systemic cancer therapy. These interactions likely also underlie the phenomenon of a mixed clinical response to therapy, where some metastases regress and others grow in response to the same treatment [3].

Over the last decade, the influence of host immune cells both within and surrounding tumors has emerged as a critical determinant of cancer biology, and a key factor in the success or failure of human cancer therapy [4]. Recognition of their impact has produced two major initiatives in the field of cancer immunotherapy: (1) the development of the "immune score" as a new and important component of the routine diagnostic and prognostic evaluation of human cancers [5], and (2) the development of combinatorial immune-based therapies that abrogate tumor-associated immune suppression in order to unleash the full tumoristatic and tumoricidal activity of pre-existing or therapeutically-induced immunity [6]. A deeper understanding of how cellular and molecular interactions within the tumor microenvironment sculpt the activities of innate and antigen-specific immune cells will lead to integrative cancer immunotherapies that selectively impinge on regulatory mechanisms within the tumor microenvironment to result in immune-based tumor rejection and clinical cure. To focus attention on these issues, the Society for the Immunotherapy of Cancer (SITC) is holding a workshop entitled "Focus on the Target: The Tumor Microenvironment" from October 24-25, 2012 in Bethesda, Maryland [7].

## The interplay of immunity and cancer growth and progression

The Janus face of the immune system in cancer presents a complex challenge for tumor immunotherapy. Cells of the innate and acquired immune systems are involved in cellular transformation, in the establishment and growth of tumors, and in the metastasis of malignant tumor cells. Carcinogenesis results from the inflammation associated with a variety of chronic infections [8]. Cells of the immune system facilitate tumor progression and spread by selecting for tumor cells intrinsically capable of escaping immune recognition [4], by creating a tumor microenvironment that fosters disease progression [4], and by facilitating the local invasion and subsequent metastasis of tumor cells [9]. Conversely, cells of the innate and acquired immune systems can protect patients against both nascent and established cancers, either by destroying cancer cells directly, or by establishing and maintaining a state of tumor dormancy [4]. The influence of the immune system on the natural history of cancer is further highlighted by observations that the concentration of CD8^+ ^T cells determines their killing efficiency in preclinical models [10], and that the quantity, quality, and location of tumor-infiltrating lymphocytes (TIL) are predictive of patient survival in cancer patients [11-14]. For example, in colon cancers, the density of CD8^+ ^T cells within the tumor predicts patient survival [11-13]. Furthermore, for many cancers including colon cancer, the relative quantity and location of CD8^+ ^T cells and CD4^+^CD25^+^FoxP3^+ ^regulatory T cells (Treg) are also key predictors of clinical outcome [12,13]. Genomic profiling of these "good prognosis," lymphocyte-infiltrated tumors typically reveals a striking signature of Th-1-type inflammation that includes markers of innate immune cell activation, chemokines that promote T cell trafficking into the tumor parenchyma, and expression of pro-inflammatory cytokines [13,15-19]. In parallel, a growing literature illustrates a role for the immune system in the clinical response to standard systemic cancer therapy. Individuals with breast cancer who carry a specific mutation of toll-like receptor 4 (TLR-4) have a higher risk of relapse after adjuvant anthracycline-based chemotherapy [20]. Patients with early breast cancers who are treated pre-operatively with paclitaxel have new immune cell infiltrates within their tumors at the time of surgery [21]. Similarly, patients treated with Trastuzumab for breast cancer can develop tumor-specific CD4^+ ^T lymphocytes within the peripheral blood [22], and within the breast tumor itself [23].

## The immune score

An immune score that quantifies the intra-tumoral location and density of CD8^+ ^T cells and memory CD45RO^+ ^T cells has been proposed as a useful approach both for predicting the impact of the tumor microenvironment on clinical outcome in colon cancer patients, and possibly for selecting therapy [13]. The clinical relevance of the immune score is that the intra-tumoral location and density of CD8^+ ^T cells and memory CD45RO^+ ^T cells are tightly correlated with disease-free and overall survival, and are, in fact, superior to the standard TNM staging system. Detailed analysis of colon cancer-associated lymphocytes reveals that a T helper type 1 profile is associated with a favorable prognosis, whereas a T helper type 17 profile is associated with a poor prognosis [24].

Other factors within the tumor microenvironment are also likely to influence the immune score, including other immune cells (intra-tumoral Treg, myeloid-derived suppressor cells, alternatively activated macrophages), stromal factors (fibroblasts, other mesenchymal cells, secretory products like tenascin [25]), and the integrity of the tumor cell genome. Of these factors, intra-tumoral Treg are one variable that has been associated with poor prognosis in many solid tumors (ovarian, breast, and pancreatic cancers), but paradoxically with favorable prognosis in colon cancer [26]. Whether cancer cell-specific genomic instability, associated with a more favorable prognosis in ovarian and colorectal cancers, is associated with or independent of the immune score remains to be determined [26,27]. Other elements of the tumor microenvironment also may shape the immune score [28]. For example, in lung cancer, low vascular endothelial growth factor-A (VEGF) and VEGF Receptor-2 expression in association with high concentrations of intra-tumoral CD4^+ ^and CD8^+ ^T cells is associated with a favorable prognosis [29].

Overall, the association of large numbers of tumor-associated CD8^+^, CD45RO^+^, and granzyme B^+ ^T cells with improved clinical outcome suggests that these cells represent the cumulative interactions of diverse tumor and host-derived cells within the tumor microenvironment. A major initiative to measure and incorporate the numbers of relevant cytotoxic memory CD8^+ ^T cells (CD8^+^, CD45RO^+^, and granzyme B^+ ^T cells) into standard clinical practice as a tumor immune score is underway [5]. A concept (based on the task force meeting "Immunoscoring as a New Possible Approach for the Classification of Cancer" convened in Naples, Italy February 13, 2012) will be presented at the SITC Workshop on the Tumor Microenvironment in October 2012. This will be followed by a "Workshop in Immune Scoring" in Naples, Italy in December 2012 that will recommend approaches to the harmonization of methods for immune scoring of tumors, and seek acceptance and implementation of immune scoring as a standard practice in the diagnosis and classification of cancers.

## Re-sculpting the tumor microenvironment to promote tumor rejection

Given the influence of the tumor microenvironment on cancer biology and overall clinical outcomes, cancer therapies that target host elements involved in cancer development are an increasingly important component of the standard of care for many cancer types. Agents that modulate the tumor microenvironment in wide clinical use today include therapeutic monoclonal antibodies that promote antibody-dependent cellular cytotoxicity (Trastuzumab for breast and gastric cancers and Rituximab for hematologic malignancies), drugs that target tumor neovascularization (bevacizumab, sunitinib, and sorafenib for a variety of cancers), and those that modify the bone microenvironment (the bisphosphonate zolendronate and the RANKL inhibitor denosumab for malignant bone disease).

There is increasing evidence that Trastuzumab [22], Rituximab, and other therapeutic monoclonal antibodies stimulate clinically relevant adaptive immune responses, and that they do so in part by cross-priming immune cells within the locoregional tumor microenvironment [30,31]. Trastuzumab-like monoclonal antibodies can promote the evolution of a tumor-specific central memory CD8^+ ^T cell response in preclinical models [32], Bevacizumab can alleviate the dendritic cell-based immune suppression caused by VEGF [33], and sunitinib can diminish the suppressive influence of intra-tumoral myeloid-derived suppressor cells and T regulatory cells in both preclinical models [34], and in patients with renal cell carcinoma [35]. Treating established tumors with a monoclonal antibody specific for the VEGF receptor 2 as a single agent can induce tumor-specific T cell immunity associated with tumor rejection and protection from a subsequent tumor challenge in an immune competent preclinical model [36]. The bone-modifying drug zolendronate can augment the activity of dendritic cells and NK cells, thereby promoting activation of γδ and αβ T cells [37]. It may also modulate the tumoristatic and tumoricidal activity of tumor-associated macrophages [38].

Importantly, immune-modulating drugs that directly promote anti-tumor immune responses (the sipuleucel-T vaccine for prostate cancer and the immune checkpoint inhibitor Ipilimumab for melanoma) have become part of the standard of care. Building on these leads, Ipilimumab and Bevacizumab have been combined with tumor vaccines to explore whether they enhance vaccine potency [39,40]. Trastuzumab has been combined with distinct cancer vaccines to capitalize on the direct antitumor activity of the antibody as well as its ability to modulate tumor immunity by various mechanisms [41,42]. Delineation of the accessory pathways that control T cell activation has led to the development of targeted checkpoint inhibitors that can further support immune priming and T cell activity within the tumor microenvironment. Novel drugs that specifically target immune regulatory pathways (including toll-like receptor modulators [43], antibodies specific for the PD-1 pathway [44], the OX-40 pathway [45], and the CD40 pathways [46]) are a growing focus of clinical development. The impact of epigenetic therapy on tumor immunity is also an emerging area of investigation [47].

## Conclusions

The findings that the proper concentration of tumor-antigen specific CD8^+ ^T cells is required to control tumor growth and eradicate antigen-expressing tumor cells in preclinical models, and that the relative numbers and distribution of CD8^+ ^T cells in human cancers is associated with disease-free and overall survival define a target goal and biomarker for clinically effective cancer immunotherapy. To meet that goal, successful immune-based therapies will likely ultimately integrate strategies that induce, recruit, and deliver tumor antigen-specific effector cells by adoptive cellular therapy or active vaccination with approaches that maximize their antitumor activity by mitigating active pathways of immune suppression within the tumor microenvironment in a scientifically rational manner. Progress in cancer immunotherapy will also require better characterization of the tumor-homing capacities, intratumoral concentration, lifespan, and functional activity of tumor antigen-specific effector T cells. The successful clinical development of such integrative cancer immunotherapies will require novel clinical trial designs that incorporate extensive blood, tissue, and imaging correlates in order to develop strategies that predict the likelihood of tumor response to immunotherapy, and evaluate immune and clinical responses in real time by imaging or tissue sampling. The overall goal of the 2012 SITC Workshop "Focus on the Target: The Tumor Microenvironment" is to focus on distinct aspects of the host-tumor interaction, and their implications for tumor immunotherapy. By systematically applying the best science to re-shape the host-tumor interaction, we will develop personalized, integrative cancer immunotherapies capable of inducing tumor rejection and effecting cure.
